# “No weight for height” case detection strategies for therapeutic feeding programs: sensitivity to acute malnutrition and target composition based on representative surveys in humanitarian settings

**DOI:** 10.1186/s40795-021-00406-6

**Published:** 2021-02-02

**Authors:** Benjamin Guesdon, Alexia Couture, Elise Lesieur, Oleg Bilukha

**Affiliations:** 1 Action Contre La Faim - France, 14-16 Boulevard Douaumont, 75854 Paris, France; 2 Emergency Response and Recovery Branch, Division of Global Health Protection, Center for Global Health, Centers for Disease Control, 1600 Clifton Road, Atlanta, GA 30329 USA

**Keywords:** Wasting, Stunting, Survey, Nutrition, Humanitarian, RUTF, MUAC, WAZ, WHZ

## Abstract

**Background:**

One newly proposed approach to determining eligibility of children aged 6–59 months for therapeutic feeding programs (TFPs) is to use mid-upper arm circumference (MUAC) < 115 mm, bilateral oedema or Weight-for-Age Z-score (WAZ) < − 3 as admission criteria (MUAC+SWAZ). We explored potential consequences of this approach on the eligibility for treatment, as compared with the existing WHO normative guidance. We also compared sensitivity and specificity parameters of this approach for detecting wasted children to the previously described “Expanded MUAC” approach.

**Methods:**

We analyzed data from 558 population representative cross-sectional cluster surveys conducted since 2007. We retrieved all children classified as severe acute malnutrition (SAM), moderate acute malnutrition (MAM), and those who are both wasted and stunted (WA + ST), and calculated proportions of previously eligible children who would now be excluded from treatment, as well as proportions of non-malnourished children among those who would become eligible. We also analyzed the expected changes in the number and demographics (sex, age) of the selected populations of children according to the different admission approaches.

**Results:**

Both MUAC+SWAZ and Expanded MUAC case detection approaches substantially increase the sensitivity in detecting SAM, as compared to an approach which restricts detection of SAM cases to MUAC< 115 mm and oedema. Improved sensitivity however is attained at the expense of specificity and would require a very large increase of the size of TFPs, while still missing a non-negligible proportion (20–25%) of the SAM caseload. While our results confirm the sensitivity of the MUAC+SWAZ case detection approach in detecting WA + ST (over 80%), they show, on the other hand, that about half of the additional target detected by using SWAZ criterion will be neither SAM nor WA + ST.

**Conclusions:**

These results suggest that recently promoted approaches to case detection inflate TFPs’ targets through the allocation of treatment to large numbers of children who have not been shown to require this type of support, including a significant proportion of non-acutely malnourished children in the MUAC+SWAZ approach. Considering the scarcity of resources for the implementation of TFPs, the rationale of abandoning the use of WHZ and of these alternative case detection strategies need to be critically reviewed.

## Background

The latest estimates of child malnutrition produced by the United Nations agencies show that globally 6.9% or 47.0 million children under 5 years of age suffered from wasting in 2019, including 14.3 million with severe wasting [1]. A child who is moderately or severely wasted has an increased risk of death [2, 3]. Wasting is responsible for approximately one-half to 1 million deaths of children under 5 worldwide each year [4, 5].

According to WHO guidelines, children with severe acute malnutrition (SAM) should be actively detected through screening and urgently referred to specific therapeutic feeding programs (TFP) that provide intensive nutritional and medical support [6]. Children suffering from moderate wasting, or moderate acute malnutrition (MAM), should also be detected and referred to appropriate care. The WHO recommendations for MAM management focus on growth monitoring, nutritional advice and medical care [7], whereas the use of supplementary foods is only recommended in settings where the prevalence of wasting or food insecurity is high [8, 9].

Current WHO case definition for SAM use low Weight-for-Height (WHZ < -3) and/or low Mid-Upper-Arm-Circumference (MUAC< 115 mm) as independent indicators, alongside nutritional oedema [6]. For detecting children with MAM, widely accepted case definition is − 3 ≤ WHZ < -2 and/or 115 mm ≤ MUAC < 125 mm [8, 10]. It is well documented that WHZ and MUAC-based criteria do not identify the same children as acutely malnourished. In order to get a quick overview of the extent of the diagnostic discrepancy, the analysis of a large dataset of cross-sectional surveys implemented in a wide range of countries and totaling around 1.4 million children demonstrated that only a minority of all SAM children (16.5%) are displaying both MUAC< 115 mm and a WHZ < -3 at the same time [11]. Little is known about the clinical significance of this diagnostic discrepancy. Recent analyses suggest that SAM children with low WHZ have similar risk of dying to SAM children with low MUAC, and that children with both low MUAC and low WHZ, as well as those with oedema and low WHZ, have significantly higher risk of death than children that fulfill only one diagnostic criterion (low WHZ or low MUAC) [3, 12, 13]. Therefore, the general normative guidance remains to use both WHZ and MUAC independently for identification and admission to treatment of children with SAM and MAM [14].

However, abandoning the use of WHZ criterion for case finding and admission to therapeutic feeding programs has been increasingly implemented in recent years [15–17]. It is put forward as a key requirement for major simplifications of the international guidance on acute malnutrition management programs, which would make these programs feasible in the most decentralized, under-equipped and under-staffed areas, thereby increasing the scale and coverage of such programs where they are most needed. Field realities in these settings often do not provide practical ways to allow for weight and height screening in the community; and even in health facilities, use of WHZ may be unfeasible due to lack of equipment, staff, or access.

Since using a measure targeting only children with MUAC < 115 mm or oedema severely restricts children eligible for treatment, different alternative case detection approaches that do not use WHZ indicator have been proposed. The first is the “Expanded MUAC-only” approach. Under this approach, screening and admission is based solely on oedema or MUAC, yet at a higher cut-off: all children with a MUAC< 125 mm or oedema would be eligible to a treatment comprising ready-to-use therapeutic food, albeit those presenting a MUAC≥115 mm, at admission or during treatment, would be considered as MAM and would receive a lower dosage [18]. Potential consequences of this approach based on data from recent population representative surveys conducted around the globe were presented in our previous article [19].

Alternatively to expanded MUAC approach, re-analysis of community cohort data from Senegal collected over 35 years ago (in 1983–84) suggested that a combination of MUAC and underweight (weight-for-age indicator, WAZ) would work best in this population for detecting deaths associated with severe anthropometric deficits including concurrent wasting and stunting [20]. This leads to the recommendation to use WAZ in therapeutic feeding case detection [21] and to pilot screening and admission using the following case definition: MUAC< 115 mm or oedema or WAZ < -3 [22]. For brevity, in this paper we will refer to this new program eligibility approach as “MUAC and Severe Underweight” (MUAC+SWAZ).

The primary objective of this study is to explore the potential consequences of “MUAC and Severe Underweight” on changing the target of therapeutic feeding programs. Our secondary objective was to compare performances of “Expanded MUAC” and “MUAC and Severe Underweight” programs, in terms of their sensitivity, specificity and implications for program size.

## Methods

Data for these analyses were obtained from Action Contre la Faim (ACF) International network, an international humanitarian non-governmental organization that conducts multiple field nutrition surveys in humanitarian settings worldwide [23]. Surveys conducted during 2007–2018 that measured both sex, age, height, weight, oedema and mid-upper arm circumference (MUAC) in children aged 6–59 months were included. All surveys included were population representative cross-sectional two-stage cluster surveys following standard survey and sampling procedure and usually conducted at the district level [24].

Survey countries were grouped into six geographic categories: Latin America and the Caribbean; East and South Africa; Democratic Republic of Congo (DRC); West and Central Africa; East Asia and Pacific; and South Asia [25]. DRC was kept as its own category due to the large number of surveys from the country. Countries that had fewer than five surveys conducted during 2007–2018 were excluded from the analyses. We considered that fewer surveys would have too few cases of acute malnutrition to produce reliable per-country estimates. The Middle East and North Africa region was not included since none of the countries had five or more surveys conducted during the study period.

Weight-for-height (WHZ), height-for-age (HAZ) and weight-for-age (WAZ) Z scores were calculated for all children using the WHO SAS macro, which applies the WHO 2006 growth standards [26]. Children with missing data for age, sex, weight, height or MUAC and with age out of range (< 6.0 months or > =60 months) were excluded. Following WHO flagging criteria, children were also excluded if they had MUAC that fell below 70 or above 220, WHZ that fell outside of + / - 5 Z-scores, HAZ that fell outside of + / - 6 Z-scores, and WAZ were < − 6 and > + 5 Z-scores.

Acute malnutrition was defined as either by MUAC only, by WHZ only, or by both criteria (MUAC and/or WHZ). Severe malnutrition (SAM) defined by MUAC (SAMmuac) was MUAC < 115 mm and/or clinical signs of oedema, and moderate acute malnutrition (MAM) defined by MUAC (MAMmuac) was MUAC < 125 mm and > =115 mm. SAM defined by MUAC and/or WHZ (SAMall) was MUAC < 115 mm and/or WHZ < − 3 and/or clinical signs of oedema, and MAM defined by MUAC and/or WHZ (MAMall) was MUAC < 125 mm and > =115 mm and/or WHZ < − 2 and > = − 3, excluding those already defined as SAMall. “Wasting and stunting” status (WA + ST) was defined as WHZ < − 2 and HAZ < − 2. “Severe underweight” (SWAZ) status was defined as WAZ < − 3.

To explore and compare the consequences of a “MUAC and Severe Underweight” (MUAC+SWAZ) program and a “Expanded MUAC” (ExpMUAC) program on the targeting of various categories of malnourished children, we defined “target” and “supplement” categories of children that would be detected and treated by each of these programs. “Target” includes all children eligible for the program. “Supplement” includes children eligible for the program in supplement to those eligible to a restricted program using only MUAC < 115 or oedema criteria. In essence, “supplement” indicates increase in program size compared to the program admitting only SAMmuac children:

- “Target MUAC+SWAZ” – all children eligible for “MUAC and Severe Underweight” program (MUAC < 115 mm or WAZ < − 3 or oedema).
- “Supplement MUAC+SWAZ” -- children eligible for “MUAC and Severe Underweight” program except those who have MUAC < 115 mm or oedema (WAZ < − 3 and MUAC > 115 mm and no oedema).
- “Target ExpMUAC” – all children eligible for “Expanded MUAC” program (MUAC < 125 mm or oedema).
- “Supplement ExpMUAC” -- children eligible for “Expanded MUAC” program except those who have MUAC < 115 mm or oedema (115 ≤ MUAC < 125 mm and no oedema).

To assess sensitivity of programs’ inclusion criteria, we calculated proportions of SAMall and MAMall children (true positives) that would be captured by the “target” and the “supplement” in each program. Thus, sensitivity of MUAC+SWAZ program “target” and “supplement” in detecting SAMall children was calculated as:

- (children that satisfy criteria of both SAMall AND “Target MUAC+SWAZ”)/(SAMall) and
- (children that satisfy criteria of both SAMall AND “Supplement MUAC+SWAZ”)/(SAMall), respectively.

Similarly, sensitivity of ExpMUAC program “target” and “supplement”, in detecting SAMall children was calculated as:

- (children that satisfy criteria of both SAMall AND “Target ExpMUAC”)/(SAMall) and
- (children that satisfy criteria of both SAMall AND “Supplement ExpMUAC)/(SAMall), respectively.

Similar calculations were done to calculate the proportions of MAMall children captured by the “target” and the “supplement” for each program (replacing SAMall with MAMall0 in formulae above).

In order to assess the adequacy of the allocation of treatment resources to children needs, and as a surrogate for specificity, we calculated proportions of children categorized as SAMall and MAMall within “target” and “supplement” in each program. For example, for the MUAC+SWAZ program, proportions of children categorized as SAMall within “target” and “supplement” were calculated as:

- (children that satisfy criteria of both SAMall AND “Target MUAC+SWAZ”)/(“Target MUAC+SWAZ”) and
- (children that satisfy criteria of both SAMall AND “Supplement MUAC+SWAZ”)/(“Supplement MUAC+SWAZ”), respectively.

Similar calculations were done for the proportions of SAMall within ExpMUAC program “target” and “supplement”, and for the proportions of chidren categorized as MAMall within “target” and “supplement” in each program.

For MUAC+SWAZ program, we also report sensitivity to WA + ST children, calculated as: and the proportion of (children that satisfy criteria of both WA + ST AND “Target MUAC+SWAZ”)/(WA + ST).

As a proxy for specificity, we use the proportion children that are categorized as neither WA + ST nor SAM, in the “target”: (children that satisfy criteria of both NOT WA + ST AND NOT SAMall AND “Target MUAC+SWAZ”)/(“Target MUAC+SWAZ”), and similarly for the “supplement”.

For country- and region-specific analyses, we aggregated all child counts from individual surveys by country and region and then calculated the proportions described above.

Further, to compare basic demographics of children in SAMall, SAMmuac and Target MUAC+SWAZ, we calculated (1) the proportion of females and (2) the proportion of younger children (aged 6–23 months) in each of these groups. Similar demographic analysis for Expanded MUAC program is reported in our previous manuscript [19].

This study was determined as non-research by the institutional review board of the Centers for Disease Control and Prevention since it entailed secondary analysis of routinely collected programmatic data. No individual identifiers were included in the dataset used for analysis. Data were aggregated, cleaned and analyzed using SAS Version 9.4 and RStudio [27, 28].

## Results

Final analyses included 558 surveys from 22 countries, which included over 406,800 children aged 6–59 months (Table 1). Overall, 0.4% of children were flagged for outlier values of MUAC, WHZ, HAZ, and/or WAZ. The sizes of the MUAC+SWAZ target and supplement, expressed as percentages of the 6–59 months population, are described by region and country in Table 1.

**Table 1:** Description of surveys included in the sample, of the “MUAC and Severe Underweight” target, and sensitivity in detecting children combining wasting and stunting, by country and region

Region	Country	N surveys	N children	SAMmuac (%)	SAMall (%)	SWAZ (%)	WA + ST (%)	Target MUAC + SWAZ (%)	Suppl. MUAC+ SWAZ (%)	Prop. of Wa + ST captured in Target MUAC+ SWAZ (%)	Prop. of Wa + ST captured in Suppl. MUAC+ SWAZ (%)	Prop. of Target MUAC+ SWAZ that are neither SAMall nor Wa + ST (%)	Prop. of Suppl. MUAC+ SWAZ that are neither SAMall nor Wa + ST = el+ (%)
**East Asia and Pacific**	Myanmar	10	5473	2.2%	4.1%	10.0%	7.4%	10.5%	8.4%	81.1%	64.3%	31.5%	39.6%
	Philippines	5	3196	0.1%	1.2%	5.9%	3.9%	5.9%	5.8%	74.0%	72.4%	47.0%	47.8%
	**Total**	**15**	**8669**	**1.4%**	**3.0%**	**8.5%**	**6.1%**	**8.8%**	**7.4%**	**79.5%**	**66.2%**	**35.3%**	**42.0%**
**Latin America and Caribbean**	**Haiti**	**24**	**13,226**	**1.2%**	**1.8%**	**4.2%**	**2.3%**	**4.6%**	**3.4%**	**83.2%**	**54.6%**	**44.3%**	**60.5%**
**South Asia**	Afghanistan	65	48,456	2.5%	4.4%	6.6%	3.9%	8.0%	5.5%	83.2%	60.2%	34.0%	49.3%
	Bangladesh	37	18,181	0.9%	2.4%	10.0%	7.7%	10.2%	9.3%	74.3%	66.4%	38.6%	42.2%
	India	9	3860	1.4%	5.6%	19.8%	17.0%	20.2%	18.7%	82.1%	76.3%	26.1%	28.1%
	Nepal	10	5522	2.5%	4.9%	17.8%	10.7%	18.3%	15.8%	85.4%	69.8%	44.0%	51.1%
	Pakistan	19	12,851	3.6%	6.4%	13.1%	8.9%	14.1%	10.6%	85.4%	62.4%	31.8%	42.5%
	**Total**	**140**	**88,870**	**2.3%**	**4.4%**	**9.5%**	**6.4%**	**10.5%**	**8.2%**	**81.5%**	**65.0%**	**34.8%**	**44.5%**
**East and South Africa**	Kenya	36	22,304	0.7%	2.5%	3.9%	3.4%	4.3%	3.6%	68.0%	60.3%	29.0%	34.7%
	Madagascar	9	3709	2.6%	2.9%	5.9%	3.5%	6.6%	4.0%	83.2%	43.5%	37.7%	62.0%
	Somalia	7	5087	1.9%	4.5%	4.9%	3.9%	6.1%	4.2%	67.3%	55.3%	26.3%	37.7%
	South Sudan	26	15,167	1.7%	5.2%	4.4%	3.8%	5.4%	3.7%	72.7%	59.4%	15.2%	22.2%
	Sudan	41	35,170	2.1%	4.9%	8.8%	6.4%	9.4%	7.3%	80.9%	62.5%	31.6%	40.5%
	Uganda	19	21,658	1.8%	2.8%	5.5%	3.8%	6.2%	4.4%	81.9%	58.1%	33.0%	46.1%
	**Total**	**138**	**103,095**	**1.7%**	**3.9%**	**6.1%**	**4.6%**	**6.8%**	**5.1%**	**77.5%**	**60.2%**	**29.5%**	**39.2%**
**West and Central Africa**	Burkina Faso	7	5365	2.6%	4.3%	12.0%	9.1%	12.3%	9.8%	81.5%	57.8%	34.1%	43.0%
	Central African Republic	13	8640	2.3%	2.9%	8.3%	4.2%	9.0%	6.8%	87.1%	54.8%	48.3%	64.2%
	Chad	18	11,046	3.4%	6.1%	13.0%	8.7%	13.9%	10.6%	84.0%	61.9%	33.3%	43.9%
	Guinea	5	4025	2.5%	3.6%	7.5%	5.0%	8.2%	5.6%	91.1%	59.6%	31.4%	45.4%
	Mali	13	9116	1.2%	2.8%	5.7%	4.4%	6.0%	4.8%	77.4%	60.8%	28.8%	36.1%
	Niger	10	6979	2.1%	3.6%	14.6%	9.1%	14.9%	12.7%	84.1%	66.0%	43.9%	51.2%
	Nigeria	5	2642	4.0%	6.9%	10.7%	7.4%	12.1%	8.1%	82.1%	59.7%	27.9%	41.6%
	Sierra Leone	16	9759	0.7%	1.5%	3.1%	2.3%	3.5%	2.7%	78.8%	64.0%	31.9%	40.8%
	**Total**	**87**	**57,572**	**2.2%**	**3.7%**	**9.1%**	**6.0%**	**9.6%**	**7.5%**	**83.2%**	**61.1%**	**36.6%**	**47.1%**
**DRC**		**154**	**135,444**	**3.2%**	**4.4%**	**8.7%**	**4.7%**	**10.3%**	**7.1%**	**86.4%**	**60.6%**	**38.9%**	**56.4%**
**Overall Aggregate Total**		**558**	**406,876**	**2.4%**	**4.0%**	**8.1%**	**5.2%**	**9.1%**	**6.8%**	**82.3%**	**61.8%**	**35.8%**	**48.2%**

SAMmuac: MUAC < 115 mm and/or oedema

SAMall: MUAC< 115 mm and/or oedema and/or WHZ < −3

SWAZ: Severe underweight defined as WAZ < −3

WA + ST: Wasting and stunting defined as WHZ < −2 and HAZ < −2

Target MUAC+SWAZ: all children eligible for “MUAC and Severe Underweight” program defined by MUAC < 115 mm or WAZ < −3 or oedema

Suppl MUAC+SWAZ: children eligible for “MUAC and Severe Underweight” program except those who have MUAC < 115 mm or oedema > = 115 mm and no oedema

*DRC* Democratic Republic of Congo

Regional and overall totals are in bold

As shown in Tables 1 and 2, overall MUAC+SWAZ target approach (9.1%) more than doubles (2.3 times increase) therapeutic feeding program size compared to the WHO recommended target (SAMall) (4.0%). It also roughly quadruples program size based on restricted SAMmuac target (the ratio of supplement MUAC+ SWAZ to SAMmuac of 2.9 in Table 3 translates into an increase of 3.9 times). This increase in the target size comes with a relatively high sensitivity in detecting SAMall (82.8, Table 2), and WA + ST (82.3%, Table 1). This high sensitivity however is attained at the expense of specificity: 63.3% of the target is not SAM (including 35.6% of the target being MAM and 27.7% not wasted at all, Table 2) and 35.8% of the target is neither SAM nor WA + ST (Table 1). When considering the supplementary population of children (supplement MUAC+SWAZ) who would be selected to therapeutic feeding program when using WAZ < -3 as an independent indicator in addition to SAMmuac, our analysis shows that almost half of them (48.2%) would be neither SAM nor WA + ST (Table 1, last column).

**Table 2:** Increase in program size, sensitivity in detecting acute malnutrition and composition of the targets of “MUAC and Severe Underweight” and “Expanded MUAC” programs, by country and region

Region	Country	MUAC + SWAZ Program						Expanded MUAC Program				
		Ratio of Target MUAC+ SWAZ to SAMall	Proportion of SAMall captured by Target MUAC+ SWAZ (%)	Proportion of MAMall captured by Target MUAC+ SWAZ (%)	Proportion of Target MUAC+ SWAZ that are SAMall (%)	Proportion of Target MUAC+ SWAZ that are MAMall (%)	Proportion of Target MUAC+ SWAZ that are neither SAMall nor MAMall (%)	Ratio of Target ExpMUAC to SAMall	Proportion of SAMall captured by Target ExpMUAC (%)	Proportion of MAMall captured by Target ExpMUAC (%)	Proportion of Target ExpMUAC that are SAMall (%)	Proportion of Target ExpMUAC that are MAMall (%)
**East Asia and Pacific**	Myanmar	2.59	81.5%	26.9%	31.4%	44.7%	24.0%	2.83	79.7%	47.2%	27.7%	72.3%
	Philippines	4.97	86.6%	38.9%	17.4%	38.1%	44.5%	1.00	31.6%	14.1%	31.6%	68.4%
	**Total**	**2.94**	**82.3%**	**28.9%**	**28.0%**	**43.0%**	**29.0%**	**2.56**	**72.7%**	**41.8%**	**27.9%**	**72.1%**
**Latin America and Caribbean**	**Haiti**	**2.62**	**90.9%**	**27.0%**	**34.7%**	**28.0%**	**37.3%**	**2.16**	**80.3%**	**50.2%**	**27.2%**	**72.8%**
**South Asia**	Afghanistan	1.82	81.7%	22.1%	44.9%	31.6%	23.5%	2.35	71.5%	63.2%	30.2%	69.8%
	Bangladesh	4.24	83.0%	36.4%	19.5%	47.6%	32.8%	2.48	65.9%	32.7%	26.5%	73.5%
	India	3.59	83.4%	45.8%	23.2%	54.9%	21.8%	1.89	63.1%	29.3%	33.2%	66.8%
	Nepal	3.78	90.3%	43.0%	23.9%	39.0%	37.0%	2.44	81.7%	47.2%	31.9%	68.2%
	Pakistan	2.22	84.1%	31.4%	37.8%	37.8%	24.3%	2.09	74.7%	50.1%	35.5%	64.5%
	**Total**	**2.41**	**83.0%**	**30.2%**	**34.5%**	**38.6%**	**26.9%**	**2.29**	**71.8%**	**50.7%**	**31.1%**	**68.9%**
**East and South Africa**	Kenya	1.72	54.8%	14.0%	31.8%	44.5%	23.7%	1.65	47.6%	21.4%	26.8%	73.2%
	Madagascar	2.28	94.5%	26.6%	41.5%	35.3%	23.2%	3.10	94.4%	71.3%	30.5%	69.6%
	Somalia	1.36	67.6%	9.6%	49.8%	29.1%	21.1%	2.73	65.8%	50.1%	24.1%	75.9%
	South Sudan	1.03	55.6%	9.7%	54.1%	34.6%	11.3%	1.80	60.4%	32.7%	33.2%	66.8%
	Sudan	1.92	69.6%	21.2%	36.2%	39.1%	24.7%	1.91	67.4%	35.0%	33.9%	66.1%
	Uganda	2.20	83.6%	24.7%	38.0%	39.3%	22.7%	3.05	82.4%	63.4%	25.4%	74.6%
	**Total**	**1.74**	**67.4%**	**17.5%**	**38.8%**	**38.8%**	**22.3%**	**2.10**	**66.2%**	**37.5%**	**30.3%**	**69.7%**
**West and Central Africa**	Burkina Faso	2.85	93.5%	35.7%	32.8%	37.3%	30.0%	2.20	86.6%	45.0%	38.2%	61.8%
	Central African Republic	3.12	94.8%	34.1%	30.3%	32.2%	37.5%	2.98	90.4%	70.5%	27.0%	73.0%
	Chad	2.27	83.6%	32.5%	36.8%	38.4%	24.8%	2.15	79.8%	50.5%	35.4%	64.6%
	Guinea	2.26	92.5%	37.4%	40.9%	35.7%	23.4%	2.15	89.7%	58.2%	37.5%	62.5%
	Mali	2.12	77.4%	20.8%	36.7%	39.9%	23.5%	2.11	66.9%	35.5%	31.6%	68.5%
	Niger	4.08	92.6%	44.5%	22.7%	44.9%	32.4%	3.16	83.9%	56.5%	26.4%	73.6%
	Nigeria	1.75	80.2%	27.1%	45.7%	33.6%	20.7%	1.75	73.1%	47.1%	40.1%	59.9%
	Sierra Leone	2.35	80.3%	25.7%	34.5%	38.0%	27.5%	2.07	65.7%	40.6%	29.4%	70.6%
	**Total**	**2.59**	**86.3%**	**32.5%**	**33.3%**	**38.3%**	**28.3%**	**2.33**	**79.9%**	**50.1%**	**32.6%**	**67.4%**
**DRC**		**2.34**	**91.3%**	**30.2%**	**38.9%**	**30.9%**	**30.2%**	**2.38**	**84.5%**	**64.4%**	**31.0%**	**69.0%**
**Overall Aggregate Total**		**2.26**	**82.8%**	**26.5%**	**36.7%**	**35.6%**	**27.7%**	**2.29**	**76.2%**	**50.2%**	**31.0%**	**69.0%**

Target MUAC+SWAZ: all children eligible for “MUAC and Severe Underweight” program defined by MUAC < 115 mm or WAZ < −3 or oedema

SAMall: MUAC< 115 mm and/or oedema and/or WHZ < −3

MAMall: MUAC < 125 mm and > =115 mm and/or WHZ < −2 and > = −3

Target ExpMUAC: children eligible for “Expanded MUAC” program defined as MUAC < 125 mm or oedema

*DRC* Democratic Republic of Congo

Regional and overall totals are in bold

**Table 3:** Increase in program size, sensitivity in detecting acute malnutrition and composition of the supplements of “MUAC and Severe Underweight” and “Expanded MUAC” programs, by country and region

Region	Country			MUAC + SWAZ Program						Expanded MUAC Program				
		N surveys	N children	Ratio of Suppl. MUAC+ SWAZ to SAM-muac	Prop of SAMall captured by Suppl. MUAC+ SWAZ	Prop of MAMall captured by Suppl. MUAC + SWAZ	Prop of Suppl. MUAC+ SWAZ that are SAMall	Prop of Suppl. MUAC+ SWAZ that are MAMall	Prop of Suppl. MUAC+ SWAZ that are neither SAMall nor MAMall	Ratio of Suppl. Exp-MUAC to SAM-muac	Prop of SAMall captured by Suppl. Exp-MUAC	Prop of MAMall captured by Suppl. Exp-MUAC	Prop of Suppl. Exp-MUAC that are SAMall	Propof Suppl. Exp-MUAC that are MAMall
**East Asia and Pacific**	Myanmar	10	5473	3.84	27.9%	26.9%	13.6%	56.2%	30.2%	4.28	26.1%	47.2%	11.4%	88.6%
	Philippines	5	3196	62.00	78.9%	38.9%	16.1%	38.7%	45.2%	11.67	23.7%	14.1%	25.7%	74.3%
	**Total**	**15**	**8669**	**5.27**	**35.4%**	**28.9%**	**14.3%**	**51.2%**	**34.5%**	**4.46**	**25.8%**	**41.8%**	**12.3%**	**87.7%**
**Latin America and Caribbean**	**Haiti**	**24**	**13,226**	**2.72**	**20.6%**	**27.0%**	**10.8%**	**38.1%**	**51.1%**	**2.07**	**9.9%**	**50.2%**	**6.8%**	**93.2%**
**South Asia**	Afghanistan	65	48,456	2.20	24.9%	22.1%	19.9%	45.9%	34.2%	3.14	14.7%	63.2%	8.2%	91.8%
	Bangladesh	37	18,181	10.59	46.5%	36.4%	12.0%	52.1%	35.9%	5.76	29.3%	32.7%	13.9%	86.1%
	India	9	3860	13.15	58.1%	45.8%	17.4%	59.1%	23.5%	6.45	37.8%	29.3%	23.1%	76.9%
	Nepal	10	5522	6.28	38.4%	43.0%	11.8%	45.2%	43.0%	3.70	29.9%	47.2%	15.6%	84.4%
	Pakistan	19	12,851	2.98	28.4%	31.4%	17.0%	50.5%	32.5%	2.74	19.0%	50.1%	12.4%	87.6%
	**Total**	**140**	**88,870**	**3.63**	**30.9%**	**30.2%**	**16.3%**	**49.4%**	**34.3%**	**3.39**	**19.6%**	**50.7%**	**11.1%**	**88.9%**
**East and South Africa**	Kenya	36	22,304	5.13	26.8%	14.0%	18.6%	53.0%	28.3%	4.87	19.5%	21.4%	14.3%	85.7%
	Madagascar	9	3709	1.56	5.6%	26.6%	4.0%	58.0%	38.0%	2.49	5.6%	71.3%	2.5%	97.5%
	Somalia	7	5087	2.29	26.3%	9.6%	27.9%	41.9%	30.2%	5.62	24.6%	50.1%	10.6%	89.4%
	South Sudan	26	15,167	2.16	23.1%	9.7%	32.8%	50.7%	16.5%	4.54	27.9%	32.7%	18.9%	81.1%
	Sudan	41	35,170	3.54	27.3%	21.2%	18.2%	50.2%	31.6%	3.53	25.2%	35.0%	16.9%	83.1%
	Uganda	19	21,658	2.52	21.2%	24.7%	13.5%	55.0%	31.6%	3.88	20.0%	63.4%	8.3%	91.7%
	**Total**	**138**	**103,095**	**3.07**	**24.8%**	**17.5%**	**18.9%**	**51.4%**	**29.6%**	**3.94**	**23.6%**	**37.5%**	**14.1%**	**86.0%**
**West and Central Africa**	Burkina Faso	7	5365	3.79	34.1%	35.7%	15.1%	47.0%	37.9%	2.70	27.2%	45.0%	16.9%	83.1%
	Central African Republic	13	8640	3.03	17.2%	34.1%	7.3%	42.8%	49.9%	2.84	12.8%	70.5%	5.8%	94.2%
	Chad	18	11,046	3.13	28.6%	32.5%	16.6%	50.7%	32.7%	2.92	24.9%	50.5%	15.6%	84.5%
	Guinea	5	4025	2.25	22.8%	37.4%	14.5%	51.5%	33.9%	2.09	20.0%	58.2%	13.7%	86.3%
	Mali	13	9116	3.99	35.0%	20.8%	20.7%	49.9%	29.4%	3.97	24.5%	35.5%	14.6%	85.5%
	Niger	10	6979	6.05	34.6%	44.5%	9.9%	52.3%	37.8%	4.46	26.0%	56.5%	10.1%	89.9%
	Nigeria	5	2642	2.04	22.5%	27.1%	19.2%	50.0%	30.8%	2.04	15.4%	47.1%	13.1%	86.9%
	Sierra Leone	16	9759	3.73	30.8%	25.7%	16.6%	48.3%	35.1%	3.17	16.1%	40.6%	10.2%	89.8%
	**Total**	**87**	**57,572**	**3.48**	**28.6%**	**32.5%**	**14.2%**	**49.3%**	**36.5%**	**3.03**	**22.1%**	**50.1%**	**12.6%**	**87.4%**
**DRC**		**154**	**135,444**	**2.20**	**18.2%**	**30.2%**	**11.3%**	**44.8%**	**43.9%**	**2.26**	**11.4%**	**64.4%**	**6.9%**	**93.1%**
**Overall Aggregate Total**		**558**	**406,876**	**2.87**	**24.4%**	**26.5%**	**14.6%**	**48.0%**	**37.3%**	**2.92**	**17.9%**	**50.2%**	**10.5%**	**89.5%**

SAMall: MUAC< 115 mm and/or oedema and/or WHZ < −3

MAMall: MAM defined as MUAC < 125 mm and > =115 mm and/or WHZ < −2 and > = −3

Suppl. ExpMUAC: children eligible for “Expanded MUAC” program except those who have MUAC < 115 mm or oedema defined by 115 ≤ MUAC < 125 mm and no oedema

Suppl. MUAC+SWAZ: children eligible for “MUAC and Severe Underweight” program (MUAC < 115 mm or WAZ < − 3) except those who have MUAC < 115 mm or oedema

*DRC* Democratic Republic of Congo

Regional and overall totals are in bold

Tables 2 and 3 present comparisons of increases in program size, sensitivity in detecting acute malnutrition, and composition of the targets for MUAC+SWAZ (left side of the tables) and ExpMUAC (right side of the tables) approaches. Table 2 describes the overall targets identified by these approaches, while Table 3 describes the supplementary targets they identify in addition to SAMmuac children. Both approaches result in very similar (and large) increases in program size. While the MUAC+SWAZ approach shows slightly higher sensitivity in detecting SAM than ExpMUAC (82.8% vs. 76.2%), its sensitivity in detecting MAM is about twice lower than for ExpMUAC (26.5% vs. 50.2%). Only about one-third of MUAC+SWAZ and ExpMUAC targets are SAM cases (36.7 and 31% respectively) (Table 2).

**Fig. 1:**
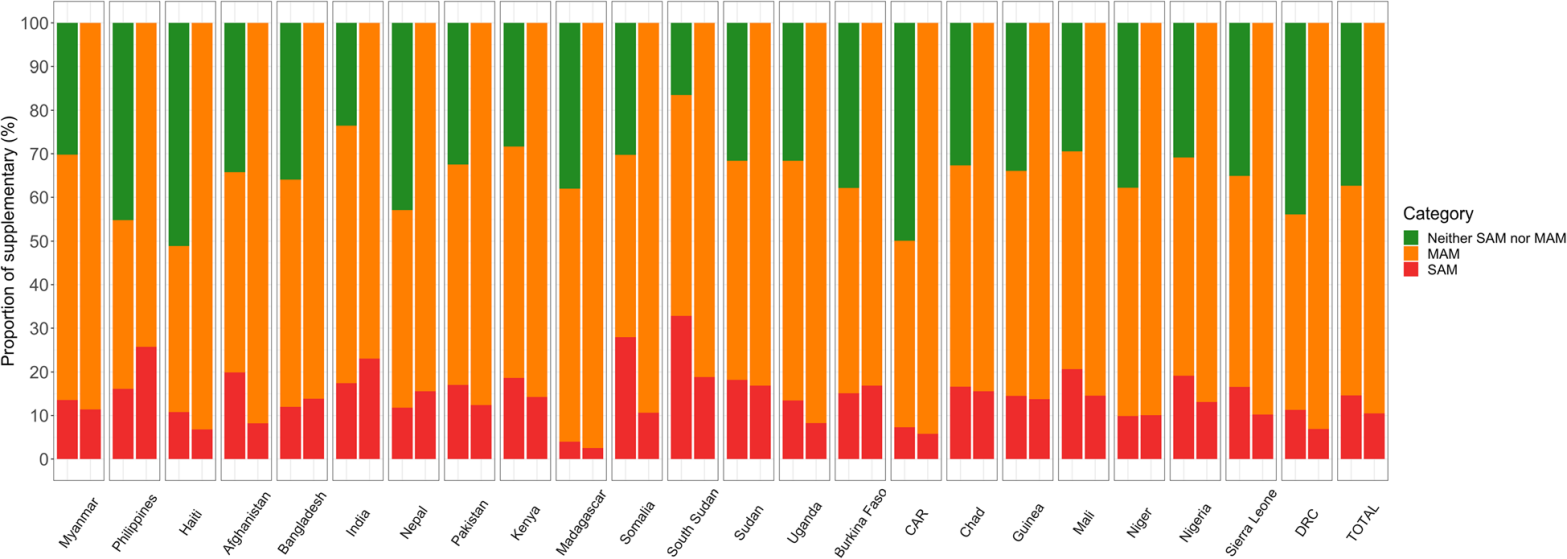
Distribution of SAM, MAM, and neither SAM nor MAM children in the supplements of “MUAC+SWAZ” (left) and “expanded MUAC” programs (right), by country. Suppl. ExpMUAC: children eligible for “Expanded MUAC” program except those who have MUAC < 115 mm or oedema defined by 115 ≤ MUAC < 125 mm and no oedema. Suppl. MUAC+SWAZ: children eligible for “MUAC and Severe Underweight” program (MUAC < 115 mm or WAZ < − 3) except those who have MUAC < 115 mm or oedema. SAM: Severe Acute Malnutrition; MAM: Moderate Acute Malnutrition. CAR: Central African Republic; DRC: Democratic Republic of Congo. Categories represented by colors are as follows: SAM (red), MAM (orange), and neither SAM nor MAM (green). Each country has a histogram on the left that represents the MUAC+SWAZ supplement and one on the right that represents the Expanded MUAC supplement

**Fig. 2:**
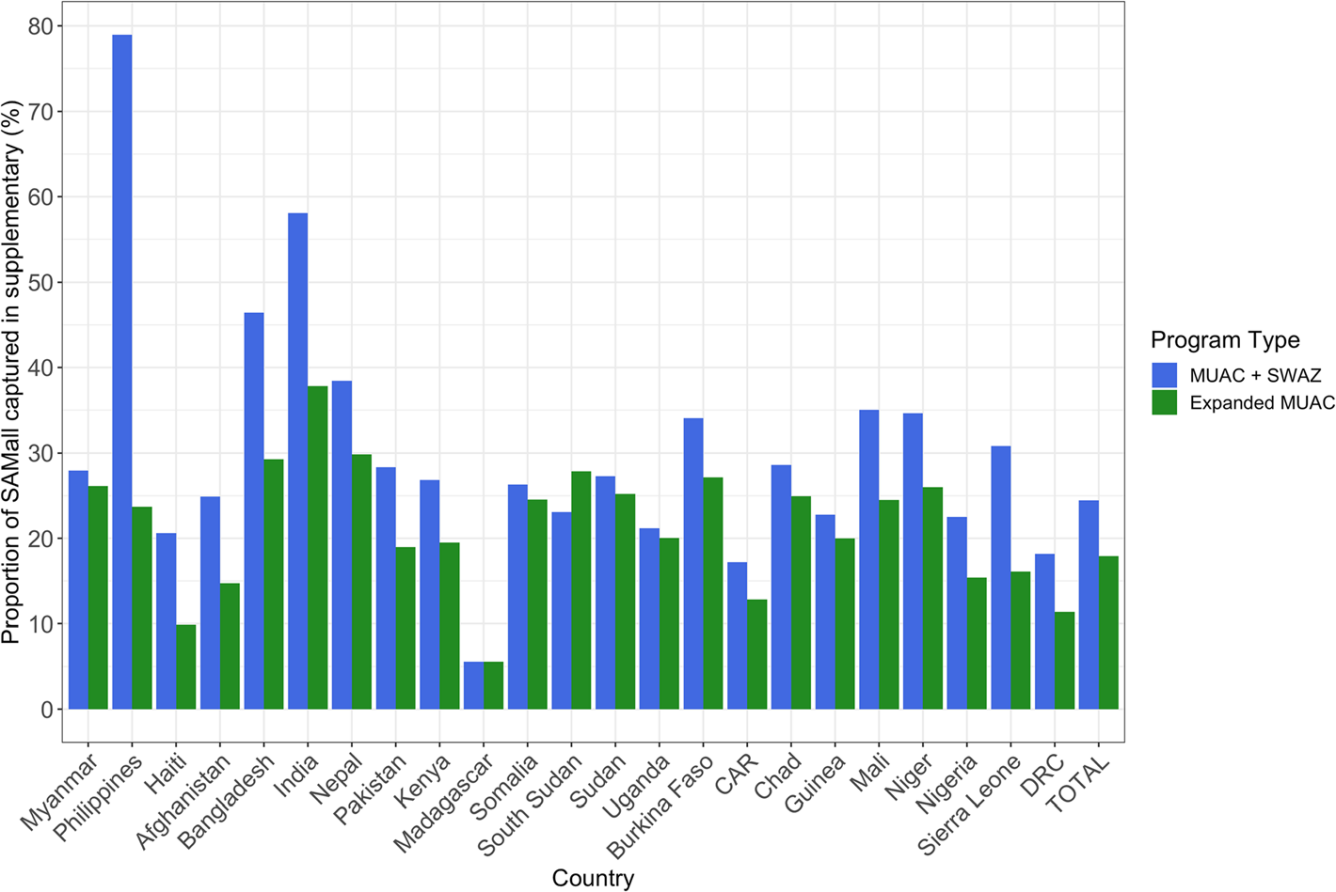
Percentage of SAMall children captured in the supplements of “MUAC +SWAZ” and “Expanded MUAC”- programs, by country. SAMall: SAM defined by MUAC< 115 mm and/or oedema and/or WHZ < − 3. Suppl. ExpMUAC: children eligible for “Expanded MUAC” program except those who have MUAC < 115 mm or oedema defined by 115 ≤ MUAC < 125 mm and no oedema. Suppl. MUAC+SWAZ: children eligible for “MUAC and Severe Underweight” program (MUAC < 115 mm or WAZ < − 3) except those who have MUAC < 115 mm or oedema. CAR: Central African Republic; DRC: Democratic Republic of Congo. Program type represented by colors are as follows: MUAC+SWAZ (blue) and MUAC Expanded (green)

If the supplemental populations of cases detected by these two approaches were to be admitted to therapeutic feeding, our results demonstrate that, depending on context, only 10–15% of them would be SAM cases (Table 3, Fig. 1). Under ExpMUAC approach, the rest of the supplemental caseload would consist of MAM children (89.5% of MAM, Table 3). Under MUAC+SWAZ approach, it would also comprise a large proportion (37.3%) of non-acutely malnourished children (i.e., children that are neither SAM nor MAM). At the same time, these supplementary targets capture relatively small proportion (less than one quarter) of all SAM cases (Fig. 2).

These results are broadly consistent across regions and countries, although higher increases in program size due to the addition of SWAZ supplement are observed in countries with much higher stunting prevalence and thus higher stunting-mediated SWAZ prevalence: Philippines (a clear outlier due to the very low number of SAMmuac cases), India, Bangladesh, and to a lesser extent Nepal and Niger. In these same countries, sensitivity of the SWAZ supplement in detecting SAM is also higher due to the large size of the supplement caseload.

Proportions of children under 2 years of age and females within classical SAMall, restricted SAMmuac and newly proposed MUAC+SWAZ targets are shown by region and country in Table 4. A consistent pattern observed across all regions is that, as compared to the classical therapeutic feeding target SAMall, the MUAC+SWAZ approach will comprise a slightly lower proportion of girls and a lower proportion of young children (< 2 years), whereas ExpMUAC target has the highest proportion of girls and children under 2 years of age.

**Table 4:** Proportion of females and children under 2 years of age in SAMall, SAMmuac, and target of “MUAC and Severe Underweight” program, by country and region

Region	Country	Proportion of SAMall that are female	Proportion of SAMmuac that are female	Proportion of Target MUAC+ SWAZ that are female	Proportion of SAMall that are < 2 years	Proportion of SAMmuac that are < 2 years	Proportion of Target MUAC+ SWAZ that are < 2 years
East Asia and Pacific	Myanmar	48.2%	61.3%	51.6%	61.3%	79.8%	43.8%
	Philippines	44.7%	33.3%	47.6%	50.0%	100.0%	31.2%
	**Total**	**47.7%**	**60.7%**	**50.6%**	**59.6%**	**80.3%**	**40.7%**
Latin America and Caribbean	**Haiti**	**49.8%**	**54.3%**	**42.1%**	**60.5%**	**64.6%**	**40.8%**
South Asia	Afghanistan	51.3%	60.2%	47.4%	72.5%	84.1%	55.7%
	Bangladesh	49.7%	65.0%	50.5%	59.3%	88.8%	35.9%
	India	43.8%	61.8%	47.8%	50.2%	81.8%	35.3%
	Nepal	48.5%	61.2%	48.3%	68.3%	85.6%	40.1%
	Pakistan	48.8%	58.1%	46.5%	65.9%	82.7%	46.8%
	**Total**	49.9%	**60.2%**	**48.0%**	**68.1%**	**84.2%**	**46.6%**
East and South Africa	Kenya	**45.4%**	57.3%	44.4%	38.1%	81.5%	36.7%
	Madagascar	47.2%	49.0%	44.3%	73.1%	80.2%	53.3%
	Somalia	46.5%	56.4%	45.3%	63.6%	88.3%	54.0%
	South Sudan	46.9%	58.1%	44.5%	52.6%	79.5%	50.2%
	Sudan	42.4%	55.8%	44.0%	58.0%	81.7%	49.6%
	Uganda	51.2%	58.7%	45.0%	71.3%	80.3%	58.3%
	**Total**	**45.4%**	**56.6%**	**44.4%**	**56.9%**	**81.3%**	**49.9%**
West and Central Africa	Burkina Faso	38.8%	54.3%	43.6%	73.7%	76.1%	49.8%
	Central African Republic	50.0%	53.1%	45.6%	55.6%	59.3%	32.4%
	Chad	51.3%	60.5%	48.2%	61.7%	74.5%	44.8%
	Guinea	49.7%	54.5%	47.6%	78.6%	85.1%	58.8%
	Mali	38.9%	59.6%	42.8%	66.9%	80.7%	51.3%
	Niger	46.1%	59.2%	46.2%	71.3%	83.0%	48.0%
	Nigeria	51.1%	57.1%	47.6%	62.1%	73.3%	44.2%
	Sierra Leone	40.6%	43.7%	38.1%	69.2%	83.1%	55.7%
	**Total**	**46.8%**	**56.7%**	**45.7%**	**65.7%**	**75.1.%**	**46.3%**
DRC		**48.0%**	**52.1%**	**44.8%**	**55.6%**	**63.2%**	**37.2%**
Overall Aggregate Total		**47.7%**	**55.3%**	**45.7%**	**60.3%**	**72.6%**	**43.5%**

SAMall: MUAC< 115 mm and/or oedema and/or WHZ < −3

SAMmuac: MUAC < 115 mm and/or oedema

Target MUAC+SWAZ: all children eligible for “MUAC and Severe Underweight” program defined by MUAC < 115 mm or WAZ < −3 or oedema

*DRC* Democratic Republic of Congo

Regional and overall totals are in bold

## Discussion

Analyses of the proportions of SAM and MAM cases detected and of the composition of the target are critical to evaluating respective merits and drawbacks of the newly proposed MUAC+SWAZ and ExpMUAC case detection approaches for admission to TFPs. Our analyses demonstrate that these “No-WHZ” case detection approaches resulting in much larger treatment targets would significantly increase the sensitivity in detecting SAM, as compared to an approach which would restrict detection of SAM cases to MUAC< 115 mm and oedema. These improvements in sensitivity would, however, be attained at the expense of specificity and would require a very large increase of the size of TFPs, while still missing a substantial proportion (20–25%) of the SAM caseload.

Protocols relying on the ExpMUAC case detection approach have already been piloted in several contexts [18, 29, 30], are promoted by a number of experts [15], and have even been recently suggested by the WHO as a possible transitory adaptation to mitigate Covid-19 infection risk, when access to essential personal protection equipment and disinfecting solutions cannot be secured [31]. Protocols relying on the MUAC+SWAZ approach, to our knowledge, have just been suggested in a few published articles and are currently the subject of an on-going research program [22]. However, to our knowledge, they have been not yet been piloted in the field. Considering the scarcity of resources for the implementation of TFPs, the rationale of proposing such increases in treatment target and of allocating treatment to large numbers of children who do not require this type of support, including a significant proportion of non-acutely malnourished children in the MUAC+SWAZ approach, is doubtful at best. Identifying children with MUAC below a higher cut-off (125 mm) or a SWAZ status could however be considered as potentially useful first-stage screening step. It would increase the opportunities for SAM and MAM case detection and could be incorporated into existing service delivery platforms: (1) active community screening using MUAC indicator, and (2) growth monitoring and promotion programs using weight-for age indicator. However, using these strategies would require the second screening step: identifying SAM cases among those selected using these screening approaches at the TFP delivery sites, and this would require using both MUAC and WHZ criteria alongside bilateral pitting oedema. Considering the high proportion of non-SAM children among the initially referred cases, these strategies would also require program enhancements to compensate the families for the referral opportunity costs (to address the cases referred by initial screening but rejected at the TFP admission site) and ensure that the whole process of coming to the TFP delivery sites for case confirmation is beneficial to them, even if children are not admitted to TFP. Such mitigation of a likely rejection effect should be achieved through adequate enhancement in programming of preventative health, nutrition and other related services which may target referred children and their families.

One of the arguments in favor of using severe underweight as an independent criterion for admission to TFPs is its sensitivity in detecting WA + ST. Our results show that the sensitivity in detecting WA + ST is indeed rather high (over 80%) under the MUAC+SWAZ case detection approach. On the other hand, about half of the supplementary target detected by using SWAZ criterion will be neither SAM nor WA + ST, which would result in inadequate use of treatment resources even assuming that WA + ST children need TFP treatment.

Further, although the health status of children that are both wasted and stunted is raising legitimate concerns [32], the suggestion that stunting significantly increases mortality risk in wasted children and thus WA + ST children should be prioritized for TFP treatment is not supported by consistent empirical evidence and requires further careful review. Elevated mortality risk in WA + ST children compared to those wasted but not stunted was reported by MacDonald and co-authors in their 2013 paper [33], yet recent evidence is challenging this paradigm [3, 34]. Indeed, one possible reason is that elevated mortality risk reported by MacDonald was observed in 0–59 months age group, where children were followed from birth and likely included substantial numbers of children who were pre-term, had intrauterine growth retardation or other inherited abnormalities, hence were both wasted and stunted at birth and died before the age of 6 months. To accurately assess whether stunting poses a significant additional mortality risk in wasted children, their data need to be reanalyzed excluding children aged 0–5 months. Further, studies examining mortality risk in 6–59 months age group paint a different picture [3, 34]. Recent reanalysis of longitudinal cohort data from Nepal, Senegal and Democratic Republic of Congo collected in 1983–1992 [3] reported that SAM children who are also stunted do not have a higher case fatality rate nor a more elevated mortality risk compared to non-stunted SAM children (see supplementary table and the narrative in the Results and Discussion sections of the article in reference [3]). Another recent work by Garenne, Briend et al. [34] found that the interaction term between stunting and wasting was not significant in increasing mortality risk in children aged 6–59 months in the Senegal 1983–84 cohort, indicating that mortality risk in wasted children did not change significantly based on whether they were stunted or not. This study also reported that mortality of children who are below − 4 combined z score, computed as (WHZ + HAZ)/2, is 11.1 times higher than in non-malnourished children. This impressive figure is of little relevance to the WA + ST discourse, since to satisfy these criteria the child has to be both extremely wasted and extremely stunted (for example, the child should have both WHZ and HAZ of − 4, which is extremely rare). This condition is very different from the WA + ST criterion, where the child has WHZ and HAZ below − 2.

Although adoption of case detection criteria that require neither height and weight measurements nor z-score assessment may address some of the barriers faced by international and national program implementers in resource constrained settings, these approaches according to our analysis would induce massive increases in program target due to loss of specificity for SAM. Adverse consequences for coverage and effectiveness of treatment for those most in need also should be expected. “No-WHZ” approaches should thus be cautiously considered only as a temporary alternative under exceptional circumstances where assessing WHZ is impossible. Under such scenario, stakeholders should be aware of the fact that any of these “no-WHZ” approaches will require large increases in program resources in order to reach the much larger targeted population. Large increase in resources does not only mean increase in funding and supply of therapeutic foods. It also means finding many additional highly qualified and trained program staff, which may be impossible in these settings irrespective of funding. Admitting and treating children that are not wasted will pose challenges and cause confusion with defining treatment progress and discharge criteria, and it will have impact on program effectiveness measures overall.

This study is based on a large number of cross-sectional population representative surveys carried out diverse humanitarian settings around the globe. Each of these surveys was planned, conducted and supervised according to the same standardized methodology, and incorporated rigorous quality controls [24]. However, these surveys were only conducted in countries where acute malnutrition is of concern to public health authorities, and where nutritional support programs were either considered or already implemented. Thus, only 22 countries (those for which we had at least 5 surveys) are represented in this analysis. In addition, surveys were mostly conducted at the district level, with the aim of providing an accurate estimate of wasting for a particularly vulnerable population in relatively small geographic area within a country. The quantitative results by country and by region are therefore provided for illustration only: these data cannot be considered as representative of the situation at country or regional level. We also observed considerable variability of results within countries and regions, therefore the overall aggregated results reported here should be interpreted with caution.

## Conclusion

Considering the scarcity of resources for the implementation of TFPs in often fragile and already overburdened health systems, the rationale of “no-WHZ” case detection strategies leading to increase their targets by several times needs to be critically reviewed. All the more so when this increase results in allocating treatment to large numbers of children who do not require this type of support, while still missing a substantial proportion of the SAM caseload.

Initially promoted for its sensitivity in detecting WA + ST, the MUAC+SWAZ approach, in particular, would include in the target a significant proportion of non-wasted children and a high proportion of children who are neither SAM nor WA + ST. It is important to reiterate that there is a contradictory evidence and no consensus on whether stunting adds a significant risk of death in wasted children after 6 months of age.

In the light of these issues, the rationale for re-investing resources in the use of WHZ for adequate case confirmation, treatment allocation, and monitoring, should be considered with more attention.

## Data Availability

The data that support the findings of this study are available from Action Contre la Faim but restrictions apply to the availability of these data, which were used under license for the current study, and so are not publicly available. Data are however available from the authors upon reasonable request and with permission of Action Contre la Faim.

## Abbreviations

ACF: Action Contre le Faim
DRC: Democratic Republic of Congo
HAZ: Height-for-age z-score
MAM: Moderate Acute Malnutrition
MUAC: Mid-upper arm circumference
RUTF: Ready-to-Use-Therapeutic-Food
SAM: Severe Acute Malnutrition
SWAZ: Severe Underweight by WAZ < − 3
TFP: Therapeutic Food Program
WA + ST: Wasting and stunting (WHZ < − 2 & HAZ < − 2)
WHO: World Health Organization
WHZ: Weight-for-height z-score
